# Feasibility and Short-Term Outcomes of Three-Dimensional Hand-Sewn Esophago-Jejunal Anastomosis in Completely Laparoscopic Total Gastrectomy for Cancer

**DOI:** 10.3390/cancers13184709

**Published:** 2021-09-20

**Authors:** Alexandros Charalabopoulos, Spyridon Davakis, Panorea Paraskeva, Nikolaos Machairas, Alkistis Kapelouzou, Ali Kordzadeh, Panagiotis Sakarellos, Michail Vailas, Efstratia Baili, Christos Bakoyiannis, Evangelos Felekouras

**Affiliations:** 1 Upper Gastrointestinal and General Surgery Unit, First Department of Surgery, Laiko General Hospital, National and Kapodistrian University of Athens, 11527 Athens, Greece; acharalabopoulos@yahoo.com (A.C.); pparaskeua@gmail.com (P.P.); akapel@bioacademy.gr (A.K.); panagiotissakarellos@gmail.com (P.S.); mike_vailas@yahoo.com (M.V.); ebaili@med.uoa.gr (E.B.); cbakoyiannis@med.uoa.gr (C.B.); felek@med.uoa.gr (E.F.); 2 Second Department of Propaedeutic Surgery, Laiko General Hospital, National and Kapodistrian University of Athens, 11527 Athens, Greece; nmachair@gmail.com; 3 Department of Surgery, Broomfield Hospital, NHS Trust, Essex CM1 7ET, UK; Alikordzadeh@gmail.com

**Keywords:** gastric cancer, laparoscopic total gastrectomy, 3D-assisted laparoscopic, hand-sewn esophago-jejunal anastomosis, outcomes

## Abstract

**Simple Summary:**

Laparoscopic total gastrectomy for the treatment of gastric and esophago-gastric junction cancer is on the rise. The procedure’s rate-limiting step is the construction of the esophago-jejunal anastomosis. Most surgeons are performing the anastomosis laparoscopically by utilizing an endoscopic linear or circular stapler; scarce evidence exists regarding laparoscopic hand-sewn esophago-jejunal anastomosis. Herein, we present our technique and results of laparoscopic manual esophago-jejunal anastomosis during totally laparoscopic total gastrectomy for gastric and esophago-gastric junction cancer. Anastomosis was performed in a two-layer fashion. Overall implementation provided excellent surgical outcomes in our cohort of patients; median anastomotic time was 55 min, while median operating time was 240 min. Intra-operative methylene blue leak test was negative in all cases. No anastomotic leak or anastomotic stricture were noted postoperatively. The 30-day and 90-day mortality rates were zero.

**Abstract:**

Laparoscopic total gastrectomy is on the rise. One of the most technically demanding steps of the approach is the construction of esophago-jejunal anastomosis. Several laparoscopic anastomotic techniques have been described, like linear stapler side-to-side or circular stapler end-to-side anastomosis; limited data exist regarding hand-sewn esophago-jejunal anastomosis. The study took place between January 2018 and June 2021. Patients enrolled in this study were adults with proximal gastric or esophago-gastric junction Siewert type III tumors that underwent 3D-assisted laparoscopic total gastrectomy. A hand-sewn esophago-jejunal anastomosis was performed in all cases laparoscopically. Forty consecutive cases were performed during the study period. Median anastomotic suturing time was 55 min, with intra-operative methylene blue leak test being negative in all cases. Median operating time was 240 min, and there were no conversions to open. The anastomotic leak rate and postoperative stricture rate were zero. The 30- and 90-day mortality rates were zero. Laparoscopic manual esophago-jejunal anastomosis utilizing a 3D platform in total gastrectomy for cancer can be performed with excellent outcomes regarding anastomotic leak and stricture rate. This anastomotic approach, although technically challenging, is safe and reproducible, with prominent results that can be disseminated in the surgical community.

## 1. Introduction

Gastric cancer (GC) is one of the most common malignancies worldwide and it is the fourth leading cause of cancer-related morbidity [1]. Despite advances in treatment, it continues to present an unfavorable prognosis, with a 5-year survival rate of around 25% across all stages [2]. A major key point in the treatment algorithm is the implementation and evolution of minimally invasive procedures over the past two decades, alongside the improvement in peri-operative chemotherapy regimens.

Laparoscopic total gastrectomy for the treatment of gastric and esophago-gastric junction cancers is on the rise. The first publication in the English literature of laparoscopic surgery for gastric cancer was in 1991 by Ohgami et al., who first reported a laparoscopic wedge resection using a lesion-lifting method [3]. Goh et al. performed the first laparoscopic-assisted distal gastrectomy with Billroth II reconstruction in 1992 [4], followed by Azagra et al. in 1996, who performed the first laparoscopic-assisted total gastrectomy for gastric cancer, with construction of the esophago-jejunal anastomosis through a mini-laparotomy [5].

Technical development of laparoscopic instruments and optics together with the growing surgical experience over time, through benign and malignant upper gastrointestional procedures, have led more surgeons and centers performing complex operations, like laparoscopic gastrectomies for cancer [6,7,8]. Despite the learning curve remaining high for such procedures, when it is reached, excellent clinical and oncological outcomes can be obtained [9]. Safety and reproducibility have been well established, as shown by the improved short-term surgical outcomes like length of hospital stay and postoperative complications with no compromise in the oncological long-term results.

With current evidence, laparoscopic total gastrectomy for proximal gastric and gastroesophageal junction cancer is considered a more advanced procedure compared to open [10]. Classically, a totally laparoscopic total gastrectomy follows the same principles as open surgery. In totally laparoscopic total gastrectomy (TLTG), the technical difficulty and the procedure’s rate-limiting step is the restoration of gastrointestinal tract continuity, with the construction of the esophago-jejunal (EJ) anastomosis [11,12,13]. While in the literature multiple anastomotic techniques have been proposed, including mechanical either with a circular or linear stapler or manual (hand-sewn) anastomosis, worldwide, none has gained popularity to be named as the gold-standard way of reconstruction. In most series though, the preferred way of reconstruction is with the use of linear staplers [14].

The aim of this study was to describe our technique of laparoscopic hand-sewn esophago-jejunal anastomosis in total gastrectomy for cancer, in a cohort of consecutive cases alongside the evaluation of clinical and histopathological outcomes.

## 2. Materials and Methods

Analysis of prospectively collected data regarding 3D-assisted laparoscopic hand-sewn esophago-jejunal anastomosis performed during TLTG for cancer was performed. Operations were performed by a single upper gastrointestinal surgeon (AC) from January 2018 to June 2021. All procedures were identical in terms of surgical team, operating surgeon, patient positioning, surgical approach, extent of lymphadenectomy and EJ anastomosis performed. This anastomotic technique has not been compared to previous ones, such as construction with a linear or circular stapler, due to the low number of the latter methods, which would not provide a sufficient sample for a valid statistical analysis.

Study inclusion criteria were adult patients (>18 years old) with no upper age limit and proximal gastric or gastroesophageal junction Siewert type III cancers that were treated with TLTG. Patients under 18 years old or gastroesophageal junction Siewert type I–II cancers were excluded. All operations for benign disease (caustic injuries, trauma, etc.) and all emergency operations were also excluded from the study.

Staging was based on clinical assessment, upper gastrointestinal endoscopy with biopsies, computed tomography of the chest and abdomen as well as staging laparoscopy. Computed tomography positron-emission tomography (PET-CT) was selectively performed in esophago-gastric junction cancers. Treatment strategies for all patients were discussed and agreed to in a dedicated multidisciplinary team setting involving surgeons, radiologists, pathologists and medical oncologists.

The study’s primary endpoints were anastomotic leak rate and anastomotic stricture rate. Secondary endpoints were set at 30- and 90-day mortality rates. All outcomes including postoperative complications (Clavien-Dindo >III) were recorded prospectively and analyzed retrospectively.

### 2.1. Laparoscopic Gastric Mobilization and D2 Lymphadenectomy

**Figure 1:**
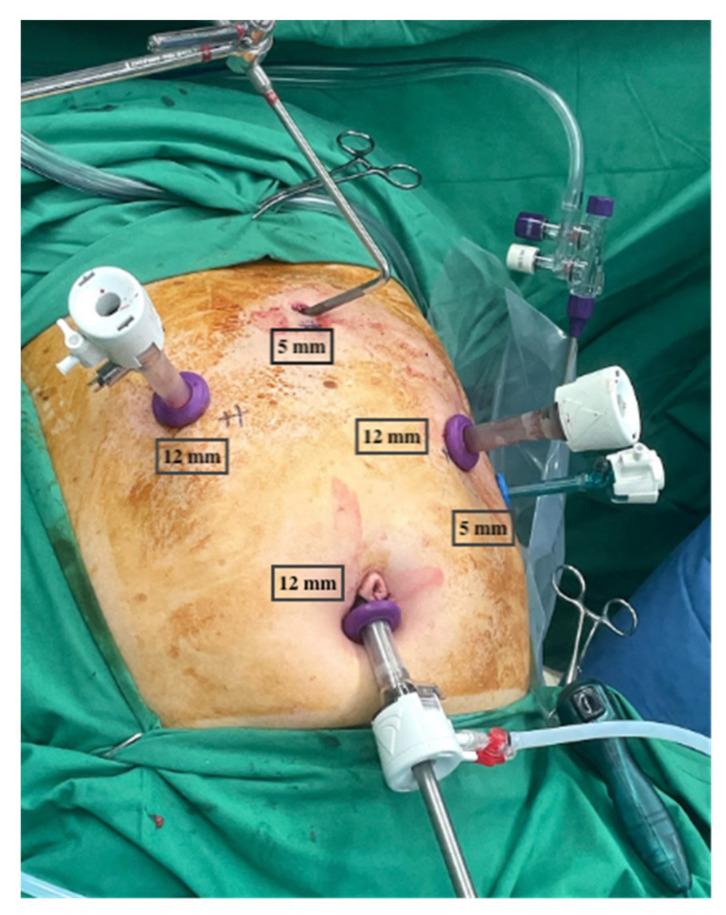
Routine trocar placement.

The patient was placed in a split-leg, steep reverse Trendelenburg position of >30^o^ with arms apart and the operating surgeon standing in between the legs, with the first assistant on the patient’s left and with the second assistant on the patient’s right side. Two 5 mm port incisions (one epigastric—for a Nathanson’s liver retractor (Mediflex, New York, NY, USA) and one left flank at the anterior axillary line—for the first assistant) and three 12mm ports (one subumbilical—camera port, one in the right mid-clavicular line—operating port, and one in the left mid-clavicular line—operating port) were placed under direct vision (Figure 1).

**Figure 2:**
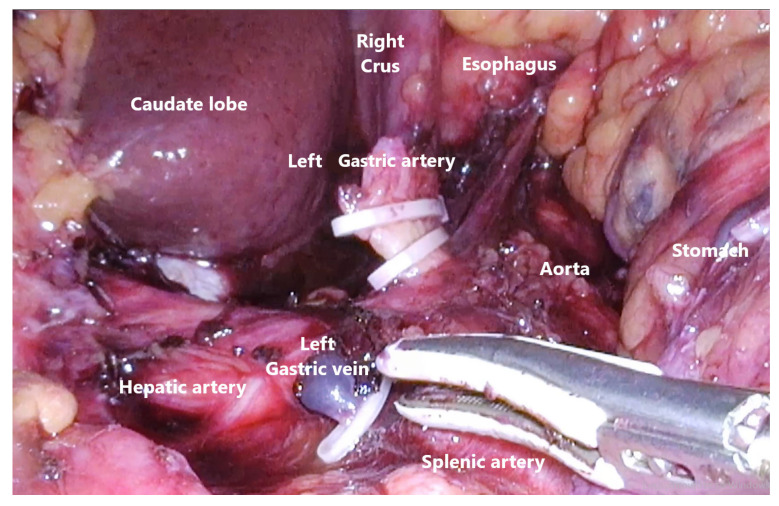
Abdominal D2 lymphadenectomy.

Routine mobilization of the stomach via division of the greater omentum from transverse colon and gastrosplenic ligament was performed with the use of an energy device (Voyant Meryland Fusion, Applied Medical, Rancho Santa Margarita, CA, USA). The lesser omentum was then dissected, starting from the duodenal bulb, following the hepatoduodenal ligament and then close to the liver edge cephalad towards the phreno-esophageal membrane, finishing on the right diaphragmatic crus. D2 lymphadenectomy with dissection of right gastric, left and right gastroepiploic, left gastric, celiac, splenic, and common hepatic nodes was performed as part of the standard procedure (Figure 2). The first part of the duodenum after full mobilization circumferentially was transected with endoscopic linear stapler with 60 mm purple cartridges (Panther, Medical Healthcare, Beijing, China).

**Figure 3:**
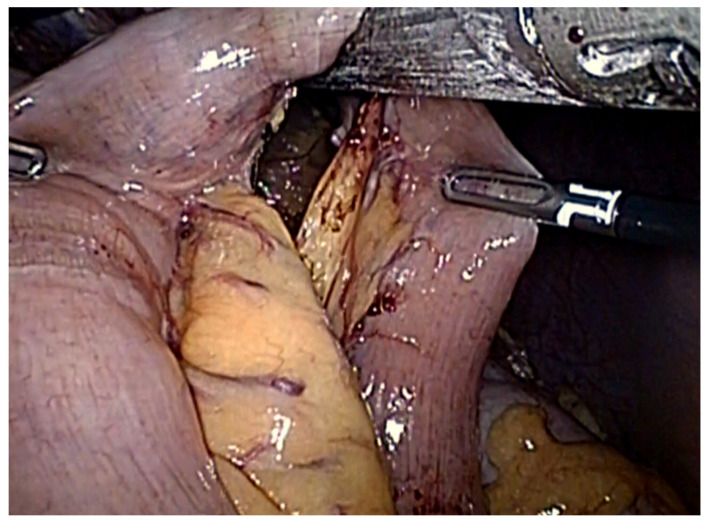
Transection of the jejunum to form the Roux limb of the esophago-jejunal anastomosis.

The esophagus was then circumferentially dissected free of surrounding tissue up to the lower mediastinum; the vagi nerves were identified and sectioned at the lower esophagus. An umbilical tape was placed around the gastroesophageal junction to help retract the stomach caudally and divide the abdominal esophagus. At this point, the cancer-free esophagus was transected with endoscopic linear stapler, with 60 mm purple cartridges (Panther, Medical Healthcare, Beijing, China). A 50 cm Roux jejunal loop was created and brought into the upper abdomen via a transmesocolic route (through an opening on the transverse mesocolon made to the left of the middle colic vessels and just cephalad of the duodeno-jejunal flexure) in preparation for the forthcoming esophago-jejunal anastomosis (Figure 3).

The surgeons’ right working port-site (in the left upper quadrant) was converted to a 4–5 cm long transverse mini-laparotomy for specimen extraction. The mini-laparotomy was then sealed using the Alexis cup system (Alexis Laparoscopic System, Applied Medical, Rancho Santa Margarita, CA, USA).

### 2.2. Laparoscopic Hand-Sewn Esophago-Jejunal Anastomosis

**Figure 4:**
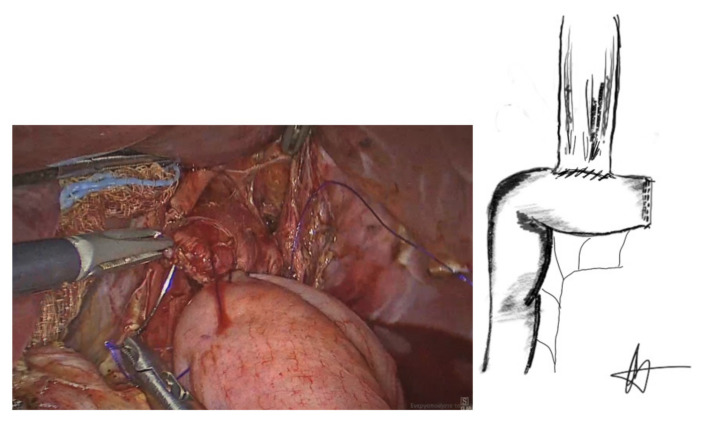
Posterior outer seromascular layer.

The anastomosis began by suturing of the esophageal staple line to the antimesenteric border of the jejunal loop with a 3/0 PDS suture (Ethicon, Somerville, NJ, USA) in an end-to-side manner, forming the posterior, outer layer of the anastomosis (Figure 4).

Anterior wall esophagotomy using hook diathermy, 1–1.5 cm above the esophageal staple line and jejunal enterotomy of equal size was created, with subsequent approximation of the two, forming the posterior inner, full-thickness layer of the esophago-jejunal anastomosis. Two 3/0 barbed sutures (STRATAFIX™ Spiral Knotless Tissue Control Devices, Ethicon, Cincinnati, OH, USA) were used for this anastomotic layer, starting at the middle of the posterior layer, and running opposite to each other to reach each side of the anterior aspect of the anastomosis.

At that stage and prior to completion of the anterior anastomotic inner layer, a naso-gastric tube was placed across the anastomosis under direct vision, in essence stenting the posterior anastomotic wall and allowing better visualization and suturing of the anterior anastomotic wall; the tip of the naso-gastric tube was left in the jejunal loop to facilitate an anastomotic methylene blue leak test at the end of the formation of the anastomosis.

**Figure 5:**
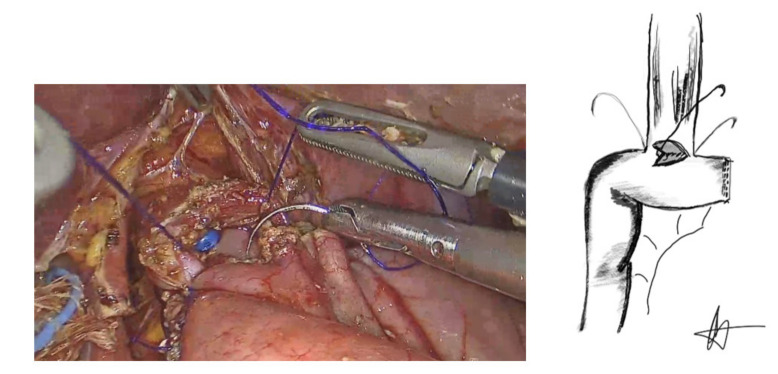
Anterior inner anastomotic layer.

At the anastomotic corners, the same barbed sutures were then reversed to provide a forehand stitch and the anterior inner anastomotic full-thickness layer was then completed (Figure 5).

**Figure 6:**
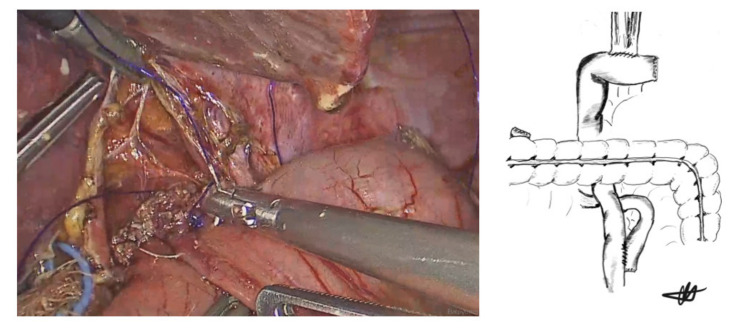
Anterior outer layer (Lembert sutures) and final view of the anastomosis.

Finally, an anterior, outer seromuscular esophago-jejunal layer using another 3/0 barbed suture (STRATAFIX™ Spiral Knotless Tissue Control Devices, Ethicon, Cincinnati, OH, USA) in a running fashion (Lembert sutures) was added to reinforce the anastomosis (Figure 6). A methylene blue leak test via the naso-gastric tube with forcep-occlusion of the Roux limb was performed in all cases. Following a negative leak test, and at the end of the procedure, the naso-gastric tube was removed (Video S1).

Subsequently, a side-to-side mechanical jejuno-jejunal anastomosis was constructed between the alimentary and biliopancreatic limbs to complete the restoration of the gastrointestinal tract. This anastomosis was performed with the use of an endoscopic linear stapler with purple cartilages of 60 mm (Panther, Medical Healthcare, Beijing, China); enterotomies were closed in a single layer, using 3/0 PDS sutures (Ethicon, Cincinnati, OH, USA).

The mini-laparotomy was closed using PDS loop sutures (Ethicon, Somerville, NJ, USA). After a final meticulous inspection of the abdominal cavity for bleeding and before releasing the carbon dioxide from the abdomen, a Robinson’s drain was placed through the left working port, across the Morrison’s space (duodenal stump), with the drain tip lying close to the newly formed esophago-jejunal anastomosis.

### 2.3. Postoperative Course

All patients were transferred to the ward postoperatively without a naso-gastric tube; they all remained nil by mouth for 72 h, when oral fluids were commenced if there was no clinical deviation from the norm. On postoperative day 6, all patients with uneventful recovery were on a soft diet and discharged a day later. A soft diet was continued for the next 30 days. Thereafter, return to normal diet was suggested in all patients.

### 2.4. Statistical Analysis

Statistical analysis was performed based on the curative intention of surgical procedures. All variables are reported as means and medians with their corresponding standard deviations and ranges. Statistical analysis was performed using SPSS software version 22.0 (IBM, Armonk, New York, NY, USA).

## 3. Results

Between January 2018 and June 2021, *n* = 40 consecutive 3D-assisted laparoscopic total gastrectomies were performed in our department by the same surgical team, for the treatment of proximal gastric and gastroesophageal junction Siewert type III tumors. All operations were identical in terms of patient positioning, port placement, extend of lymphadenectomy and laparoscopic hand-sewn construction of the EJ anastomosis.

The male-to-female ratio was 3.4/1, with a mean age of 65 years. While neoadjuvant chemotherapy was administered to 75% of patients, 86.66 % of that cohort received the full peri-operative scheme. Radiotherapy was not administered to any of the patients. Patients’ demographics and pre-operative interventions are presented in Table 1.

There was no blood loss >200 mL and there were no conversions to open. The median operative time was 240 min, while the median time taken for the construction of the EJ anastomosis was 55 min. The intra-operative methylene blue leak test was negative in all cases and no anastomotic leak was noted during the postoperative period. There were no anastomotic strictures seen and the 30- and 90-day mortality rates were zero. The operative and clinical outcomes are presented in Table 2.

Most patients had poor or moderate tumor differentiation, with tumor T-status of greater than pT2 in 52.5% of the patients; positive nodal disease was present in 65% of the cases. Median number of resected lymph nodes was *n* = 35. The histopathological outcomes are presented in Table 3.

## 4. Discussion

Laparoscopic total gastrectomy for cancer is on the rise and its utilization on upper gastrointestinal diseases has long been established, offering all the short-term advantages of minimally invasive surgery. Laparoscopic distal gastrectomy was first performed in 1992 by Goh et al. [3], while the first laparoscopic total gastrectomy was published in 1999 by Azagra et al. [4], leading to gradual implementation and adoption of laparoscopic gastrectomy for the treatment of gastric and esophago-gastric junction cancers [5,6,7].

In their studies, Xiong, J.J. et al. and Shi, Y. et al. showed that laparoscopic total gastrectomy can provide favorable clinical outcomes compared to open surgery; intra-operative blood loss, overall postoperative complication rate and length of hospital stay are reduced in a statistically significant degree with the laparoscopic approach [10,15]. Subsequently, the latter offers a favorable oncological long-term outcome as seen by markers like disease-free survival and overall survival [16,17]. Additionally, further studies of retrospective nature [18,19] as well multicenter randomized clinical trials [20,21] have shown similar results.

The rate-limiting step of TLTG is the construction of EJ anastomosis. In the first cases of LTG gastrectomy, esophago-jejunal anastomosis was performed with a circular stapler in an end-to-side manner, through a mini-laparotomy [22]. Numerous publications report the construction of the anastomosis either with a linear or circular stapler laparoscopically or through a mini-laparotomy through the left upper quadrant [23,24].

Intracorporeal construction of the EJ anastomosis via endoscopic linear stapler has been the preferred method of reconstruction by most surgeons worldwide [14]. Two approaches have mainly been performed: the functional end-to-end stapling and the overlap method [25]. The functional method presents a safe and easy method of construction of the EJ anastomosis, whereas the overlap technique requires less extensive mobilization of the jejunal limb. The overlap technique is safer and more feasible than the functional method, mainly due to significantly decreased incidence of anastomotic-related complications in published literature [26].

Multiple ways of constructing the anastomosis using circular staplers have been proposed, both intra-corporeally and extracorporeally [27]. The anvil of the circular stapler can be placed either laparoscopically through the abdomen or it may be advanced using the naso-gastric tube, also known as oral anvil or OrVil (Corviden, Mansfield, MA, USA) [28]. For the fixation of the anvil, a laparoscopic hand-sewn purse-string [29], Endostich (Corviden, Mansfield, MA, USA) [30], or an endo-purse-string instrument like Endo-PSI (Hope Electronics, Chiba, Japan) [31] have been used. Notably, utilization of the OrVil does not require purse-string suturing, as the system is designed for trans-oral insertion of the anvil into the distal esophagus, but unanimously presents a more technically challenging step of the operation for the anesthesiologist.

Overall, linear-stapled anastomosis is considered easier to perform compared to the circular-stapled anastomosis [32,33]. Another key difference between these two anastomotic techniques is the reported superiority of linear-stapled anastomosis regarding anastomotic strictures (0 versus 6–17%) [32]. Furthermore, anastomotic bleeding is reduced and in favor of the linear stapler technique (0.5% versus 5.5% with circular stapler) [14]. Likewise, decreased anastomotic leak rate is reported with the linear-stapled technique (2% versus 9% with a circular stapler) [34].

To the best of our knowledge, there is limited data regarding laparoscopic hand-sewn EJ anastomosis during TLTG for cancer; the first report was by So et al. in 2011 [34], followed by Chen et al. [28], Norero et al., Xu et al. in 2017 [35,36], and finally Yan et al. in 2020 [37]. In these published series, the anastomotic leak rate reached up to 3.8% (0–3.8%) and the anastomotic stricture rate presented as high as 3.7% (0–3.7%). Details and comparison of the studies are presented in Table 4.

This study presents our surgical technique of TLTG, utilizing hand-sewn EJ anastomosis in a two-layer fashion. Our team began to perform laparoscopic total gastrostomies for gastric and esophago-gastric junction tumors in 2013. Initially, we constructed the EJ anastomosis with a circular stapler. However, since mid-2016 and following the refinement of our laparoscopic skills coupled with the excellent results of the hand-sewn esophago-gastric anastomosis during totally minimally invasive 2-stage esophagectomy [38,39], we have routinely performed manual laparoscopic EJ anastomosis in all comers.

All operations in our cohort were performed laparoscopically using three-dimensional (3D) optics, utilizing an Endoeye 3D Video Telescope; with 30° tip (Olympus Europa SE & CO. KG, Hamburg, Germany). We have been using 3D telescopes since 2016, and we have concluded that laparoscopic view was significantly improved with the glasses-based 3D optical system using the 30° tip camera in comparison to the relevant 2D optical system. Safer and easier laparoscopic construction of the EJ anastomosis as well as more precise lymphadenectomy were made feasible, mainly due to better depth perception with the 3D camera, thus potentially implying an improvement in postoperative and oncological outcomes.

While laparoscopic EJ anastomosis is technically one of the most demanding ones to perform, we found it to be safe and reproducible in the hands of experienced laparoscopic surgeons. In the forty consecutive cases of our cohort, anastomotic leak rate, anastomotic stricture rate, 30-day and 90-day mortality rates were all zero.

The study’s limitations are its retrospective nature coming from a single institution and due to its relatively small cohort size. Further large-scale studies and preferably randomized clinical trials are needed to elucidate this anastomotic technique and to confirm its safety, efficacy and reproducibility in laparoscopic total gastrectomy.

## 5. Conclusions

Hand-sewn esophago-jejunal anastomosis during laparoscopic total gastrectomy for gastric and esophago-gastric junction cancer is technically demanding. However, it provides excellent results in the hands of experts, with lower anastomotic leak and stricture rates compared to other anastomotic techniques, and may lead to a paradigm shift. Further studies are needed to shed light on the short- and long-term outcomes and to supplementarily investigate the adoptability of the technique by a wider surgical audience.

## Figures and Tables

**Table 1:** Epidemiological and clinical characteristics of the study population.

Characteristics	Patients (*n* = 40)
Mean Age (Range)	63.2 years (Range: 32–84)
Gender Male Female	*n* = 31 (77.5%) *n* = 9 (22.5%)
Mean BMI	27.5 kg/m^2^ (Range: 23–49)
ASA score I II III IV	*n* = 5 (12.5%) *n* = 20 (50%) *n* = 12 (30%) *n* = 3 (7.5%)
Neoadjuvant chemotherapy	*n* = 30 (75%)
Adjuvant chemotherapy	*n* = 26 (65%)
Primary Tumor Location Stomach Esophago-gastric junction (Siewert type III)	*n* = 25 (62.5%) *n* = 15 (37.5%)

BMI = Body Mass Index, ASA = American Society of Anesthesiologists.

**Table 2:** Operative and clinical outcomes.

Mean Operative Time	240 min (Range: 180–300 min)
Mean anastomotic suturing time	55 min (Range: 40–80 min)
Conversion to open	0
Postoperative complications Anastomotic leak Anastomotic stricture	0 0
30-day mortality	0
90-day mortality	0
Median length of stay	8 days (Range: 7–9 days)
Median follow-up	19 months (Range: 1–38 months)

**Table 3:** Histopathological outcomes.

Tumor Differentiation Poor Moderate Well-differentiated	*n* = 15 (37.5%) *n* = 17 (42.5%) *n* = 8 (20%)
Tumor T-status pT1a pT1b pT2 pT3 pT4a	*n* = 1 (2.5%) *n* = 3 (7.5%) *n* = 15 (37.5%) *n* = 16 (40%) *n* = 5 (12.5%)
Tumor N-status N0 N1 N2 N3a N3b	*n* = 14 (35%) *n* = 10 (25%) *n* = 11 (27.5%) *n* = 2 (5%) *n* = 3 (7.5%)
Median number of lymph-nodes extracted	*n* = 35 (Range: 18–50)

**Table 4:** Comparison of previous studies.

Authors	Design	Year	Number of Patients	Anastomotic-Related Complications *
*So et al.* [34]	Retrospective	2011	6	0
*Chen et al.* [28]	Retrospective	2017	31	2 (6.45%)
*Norero et al.* [35]	Retrospective	2017	51	3 (5.88%)
*Xu et al.* [36]	Retrospective	2017	100	4 (4%)
*Yan et al.* [37]	Retrospective	2020	44	1 (2.27%)

* Complications = anastomotic leak, anastomotic stricture, bleeding.

## Data Availability

The data presented in this study are available on request from the corresponding author.

## Supplementary Materials

The following are available online at https://www.mdpi.com/article/10.3390/cancers13184709/s1, Video S1: Laparoscopic hand-sewn esophago-jejunal anastomis.
